# Functional Polyion Complex Micelles for Potential Targeted Hydrophobic Drug Delivery

**DOI:** 10.3390/molecules27072178

**Published:** 2022-03-28

**Authors:** Radostina Kalinova, Ivaylo Dimitrov

**Affiliations:** Institute of Polymers, Bulgarian Academy of Sciences, Akad. G. Bonchev St., Bl. 103-A, 1113 Sofia, Bulgaria; kalinova@polymer.bas.bg

**Keywords:** peptide-based block copolymers, synthesis, polyion complex micelles, drug delivery, curcumin

## Abstract

Polyion complex (PIC) micelles have gained an increasing interest, mainly as promising nano-vehicles for the delivery of various hydrophilic charged (macro)molecules such as DNA or drugs to the body. The aim of the present study is to construct novel functional PIC micelles bearing cell targeting ligands on the surface and to evaluate the possibility of a hydrophobic drug encapsulation. Initially, a pair of functional oppositely charged peptide-based hybrid diblock copolymers were synthesized and characterized. The copolymers spontaneously co-assembled in water into nanosized PIC micelles comprising a core of a polyelectrolyte complex between poly(L-aspartic acid) and poly(L-lysine) and a biocompatible mixed shell of disaccharide-modified poly(ethylene glycol) and poly(2-hydroxyethyl methacrylate). Depending on the molar ratio between the oppositely charged groups, PIC micelles varying in surface charge were obtained and loaded with the natural hydrophobic drug curcumin. PIC micelles’ drug loading efficiency, in vitro drug release profiles and antioxidant activity were evaluated. The preliminary results indicate that PIC micelles can be successfully used as carriers of hydrophobic drugs, thus expanding their potential application in nanomedicine.

## 1. Introduction

Supramolecular aggregates formed via self-association of block copolymers characterized by structural and functional variety have gained an increasing interest for numerous applications in the field of nanotechnology [1]. As a result, nanosized systems and devices for energy conversion, data storage, catalysis and nanomedicine have been developed [2,3,4]. Among these systems, polymer core-shell micelles formed via self-association of amphiphilic block copolymers in water play a significant role, mainly as multifunctional nanocarriers of hydrophobic drugs and/or imaging agents in their cores [5,6,7]. Other organic (lipid-based, dendrimers or polymersomes) and inorganic (quantum dots, mesoporous silica or metal) nanosystems have also been evaluated for drug delivery and diagnostic applications [8,9,10]. At a certain concentration called critical micelle concentration (CMC), the amphiphilic block copolymers start to self-assemble in aqueous media. The process is driven by nonpolar hydrophobic interactions between the lipophilic polymer segments trying to avoid contact with the hydrophilic solvent and thus forming the micelle’s core [11,12]. The nanostructures are stabilized by the hydrophilic polymer blocks that are in contact with the water molecules. The amphiphilic block copolymers intended for drug delivery applications usually consist of biodegradable hydrophobic segments and biocompatible charged or neutral hydrophilic blocks ensuring the micelles’ stability in aqueous media [13]. The advances in polymerization and modification synthetic methods allow for the design of functional micelles decorated with various biochemically active groups for targeted delivery applications [14]. The general method for preparation of polymer micelles from amphiphilic block copolymers involves their dissolution in a good-for-all-segments organic solvent followed by exchange with water [15]. Another concept of polymer micelles formation in which the driving force for self-assembly is based on electrostatic interactions between copolymers bearing oppositely charged and neutral hydrophilic segments was introduced in 1995 by Kataoka et al. [16]. In contrast to the above-described micelles’ formation from amphiphilic block copolymers, here, narrow size-distributed particles were formed spontaneously through the co-assembly of simply mixed aqueous solutions of hydrophilic charged macromolecules. They were referred to as polyion complex (PIC) micelles. Other research groups working on that topic called them block ionomer complexes (BIC) [17], complex coacervation core micelles (C3Ms) [18], or (inter)polyelectrolyte complexes, (I)PEC [19,20]. The PIC micelles are core–shell structures consisting of a polyion complex as a core and are stabilized by the hydrophilic shell formed by the neutral copolymers’ segments. Their main advantage is that they are formed spontaneously in aqueous solution under thermodynamic equilibrium conditions [21]. Besides synthetic charged block copolymers as oppositely charged counterpart various hydrophilic macromolecules such as peptides, proteins, nucleic acids or oligonucleotides could be used and thus condensed into the PIC micelles core via electrostatic interactions, making them attractive for applications in nanomedicine [22]. As a result, numerous nanosized complexes (polyplexes) between synthetic polymers and proteins [23], DNA [24] or RNA [25] have been developed and evaluated as non-viral delivery vehicles. In order to improve their efficiency, selected polyplexes were further modified to achieve enhanced stability and cellular specific targeting [26,27]. Alternatively, charged or neutral low-molecular weight hydrophilic drugs have also been encapsulated into the PIC micelles for drug delivery purposes [28,29,30,31]. Conversely, there are limited reports on the potential application of PIC micelles as nanocarriers of hydrophobic drugs. In most of them, one of the charged copolymer counterparts contains hydrophobic segments. As a result, the formed PIC micelles contain an additional hydrophobic layer for drug accommodation. Thus, hydrophobic therapeutics such as paclitaxel [32], deoxypodophyllotoxin [33] and tacrolimus (FK-506) [34] were encapsulated into PIC micelles. Another report describes the formation of PIC micelles through electrostatic interactions between poly(2-ethyl-2-oxazoline)-*b*-poly(aspartic acid) block copolymer and the amine groups of the antifungal drug amphotericin B. Due to the drug’s poor water solubility, both components were dissolved in organic solvent and the drug-loaded PIC micelles were prepared by the thin film hydration method as a result of electrostatic interactions [35]. To the best of our knowledge, there are no reports on hydrophobic drug encapsulation into prepared-in-aqueous-media PIC micelles from pair hydrophilic block copolymers bearing oppositely charged segments.

Herein, we present the synthesis and characterization of two oppositely charged peptide-based functional hydrophilic block copolymers that were used for the preparation of PIC micelles as potential drug delivery systems. Initially, poly(2-hydroxyethyl methacrylate)-*b*-poly(L-lysine) block copolymer (PHEMA-*b*-PLLys) was synthesized via controlled radical polymerization followed by ring-opening polymerization initiated by a heterobifunctional initiator. The oppositely charged poly(ethylene glycol)-*b*-poly(L-aspartic acid) was synthesized by ring opening polymerization of *β*-benzyl-L-aspartate N-carboxyanhydride initiated by heterobifunctional poly(ethylene glycol) followed by modification with a cell-targeting ligand (LBA-PEG-*b*-PLAsp). Functional PIC micelles varying in surface charge were formed through co-assembly of the two copolymers in aqueous media at different molar ratios between the charged groups. They were physico-chemically characterized and loaded with the hydrophobic model drug curcumin. The drug-loading efficiency and drug-loading capacity of the PIC micelles were evaluated. The in vitro drug release profiles and antioxidant activity of the curcumin-loaded particles were estimated as an initial evaluation of their potential application as nanocarriers for targeted drug delivery.

## 2. Materials and Methods

### 2.1. Chemicals and Reagents

All the chemicals were purchased from Sigma-Aldrich. 2-Hydroxyethyl methacrylate (HEMA, 98%) was passed through a column containing basic aluminum oxide. Aminopolyethylene glycol (HO-PEG-NH_2_, *M*_n_ = 3000 g mol^−1^) was freeze-dried from toluene. Dichloromethane (DCM, ≥99.5%), N,N-dimethylformamide (DMF, ≥99.8%), and dimethyl sulfoxide (DMSO) were purified through a distillation from calcium hydride. Copper(I) bromide (CuBr, 99.999%), 2,2′-bipyridine (bpy, ReagentPlus^®^, ≥99%), N,N′-dicyclohexylcarbodiimide (DCC, ≥99%), lactobionic acid (LBA, 97%), 2,2-diphenyl-1-picrylhydrazyl free radical (DPPH•), curcumin (Curc), 1,6-diphenyl-1,3,5-hexatriene (DPH, 98%), trifluoroacetic acid (TFA, 99%), HBr (33 wt% in acetic acid), methanol (99.8%) and diethyl ether (≥99.5%) were used as received.

The functional ATRP initiator *t*-Boc-aminoethyl 2-bromoisobutyrate (BAEBIB) was synthesized according to a previously reported procedure [36]. N^ε^-(benzyloxycarbonyl)-L-lysine N-carboxyanhydride (ZLLys-NCA) and *β*-benzyl-L-aspartate N-carboxyanhydride (BzLAsp-NCA) were prepared from Z-L-lysine and *β*-benzyl-L-aspartate, respectively, and triphosgene in ethyl acetate applying the unconventional purification technique described by Poché et al. [37]. Yield: 94% for ZLLys-NCA and 70% for BzLAsp-NCA. 4-(Dimethylamino)pyridinium p-toluenesulfonate (DPTS) was prepared from 4-(dimethylamino)pyridine and p-toluenesulfonic acid according to a literature procedure [38].

### 2.2. Synthesis of PHEMA-b-PLLys Block Copolymer

#### 2.2.1. Synthesis of Poly(2-Hydroxyethyl Methacrylate) with Protected Amine End-Group (PHEMA-N-Boc)

CuBr (0.096 g, 0.67 mmol), 2,2′-bipyridine (0.26 g, 1.67 mmol) and BAEBIB (0.21 g, 0.67 mmol) were introduced in a round bottom flask, and three vacuum/argon cycles were performed. In another flask, 2 g (1.86 mL) of HEMA (0.01538 mol) was dissolved in 2 mL of methanol, and the solution was degassed through bubbling with nitrogen for 60 min. The degassed HEMA solution was added to the first reaction flask. The reaction proceeded at 30 °C for 4 h. The polymerization was stopped in liquid nitrogen. The reaction mixture was passed through a column containing SiO_2_ followed by solution concentration. The product was precipitated in cold methanol and reprecipitated in methanol/diethyl ether mixture. Yield: 92%. ^1^H NMR (600 MHz, DMSO-d_6_, *δ*, ppm): 6.89 ((CH_3_)_3_-C-O-CO-N*H-*), 4.86 (-O*H*), 4.16 (-NH-CH_2_-C*H*_2_-O-), 3.89 (-O-C*H*_2_-CH_2_-OH), 3.57 (-O-CH_2_-C*H*_2_-OH), 3.15 (-NH-C*H*_2_-CH_2_-O-), 1.65–2.02 (-C*H*_2_-C(CH_3_)-), 1.38 ((C*H*_3_)_3_-O-CO-NH-), 1.09 (-C(C*H*_3_)_2_-), 0.76–0.94 (-CH_2_C(C*H*_3_)-).

#### 2.2.2. Synthesis of Poly(2-Hydroxyethyl Methacrylate) Macroinitiator (PHEMA-NH_2_)

To obtain PHEMA-NH_2_, 1.9 g of PHEMA-N-Boc was dissolved in 9 mL of TFA. The reaction mixture was stirred under argon for 4 h at room temperature. The mixture was then precipitated in diethyl ether and reprecipitated from methanol/diethyl ether. The product was dispersed in water, and the pH was adjusted to 9.0 by dropwise addition of 10*N* NaOH. The salts were removed via ultrafiltration, and the final product was recovered through lyophilization. Yield: 99%. ^1^H NMR (600 MHz, DMSO-d_6_, *δ*, ppm): 4.81 (-O*H*), 4.22 (H_2_N-CH_2_-C*H*_2_-O-), 3.89 (-O-C*H*_2_-CH_2_-OH), 3.57 (-O-CH_2_-C*H*_2_-OH), 3.12 (H_2_N-C*H*_2_-CH_2_-O-), 2.89 (-O-CH_2_-CH_2_-N*H*_2_), 1.65–2.02 (-C*H*_2_-C(CH_3_)-), 1.09 (-C(C*H*_3_)_2_-), 0.77–0.94 (-CH_2_C(C*H*_3_)-).

#### 2.2.3. Synthesis of PHEMA-*b*-PZLLys Block Copolymer

Initially, 1.15 g (0.34 mmol) of PHEMA-NH_2_ was dissolved in 25 mL of DMF. Separately, 3.6 g (11.8 mmol) of ZLLys-NCA was dissolved in 15 mL of DMF. Both solutions were degassed by bubbling argon for 2 h and were combined via a transfer needle. The polymerization was carried out at 60 °C for 5 days under dry argon atmosphere. The reaction mixture was concentrated under vacuum, and the product was precipitated in cold diethyl ether and dried in a vacuum oven at 60 °C. The resulting block copolymer was stirred for 1 h in a mixture of H_2_O/MeOH = 2.5:1 (*v*/*v*) to remove any unreacted PHEMA-NH_2_ macroinitiator. The white precipitate was filtered and dried in a vacuum oven at 30 °C. Yield: 97%. ^1^H NMR (600 MHz, DMSO-d_6_, *δ*, ppm): 7.82–8.21 (αCH-N*H*-), 6.85–7.41 (-αCH-(CH_2_)_4_-N*H* + -N*H*-CH_2_-CH_2_-O- + C_6_*H*_5_-), 4.98 (Z-C*H*_2_-), 4.85 (-O*H*), 3.90–4.35 (-αC*H*-NH- + -NH-CH_2_-C*H*_2_-O-), 3.86 (-O-C*H*_2_-CH_2_-OH), 3.57 (-O-CH_2_-C*H*_2_-OH), 2.80–3.15 (-NH-C*H*_2_-CH_2_-O- + -αCH-(CH_2_)_3_-C*H*_2_-), 1.05–2.10 (-αCH-(C*H*_2_)_3_-CH_2_- + -C*H*_2_-C(CH_3_)-) + (-C(C*H*_3_)_2_-), 0.75–0.91 (-CH_2_C(C*H*_3_)-).

#### 2.2.4. Primary Amine Groups Deprotection

The Z-protecting groups were removed from the peptide block of PHEMA-b-PZLLys following the general procedure: 1.6 g of the polymer (4.59 mmol Z-protecting groups) was dissolved in 12 mL of TFA. Then, 3.5 mL of HBr (33 wt% in acetic acid) was added. The reaction mixture was stirred for an hour at room temperature followed by the addition of 25 mL of chilled water. The mixture was extracted three times with dichloromethane. The aqueous layer was neutralized with 10*N* NaOH and was dialyzed against distilled water. The product was recovered through lyophilization. Yield: 55%. ^1^H NMR (600 MHz, D_2_O, *δ*, ppm): 3.95–4.45 (-αC*H*-NH- + -NH-CH_2_-C*H*_2_-O- + -O-C*H*_2_-CH_2_-OH), 3.80 (-O-CH_2_-C*H*_2_-OH), 2.93 (-NH-C*H*_2_-CH_2_-O- + -αCH-(CH_2_)_3_-C*H*_2_-), 1.25–2.30 (-αCH-(C*H*_2_)_3_-CH_2_- + -C*H*_2_-C(CH_3_)-), 1.15–0.75 (-C(C*H*_3_)_2_- + -CH_2_C(C*H*_3_)-).

### 2.3. Synthesis of LBA-PEG-b-PLAsp Functional Block Copolymer

#### 2.3.1. Synthesis of HO-PEG-*b*-PBzLAsp Block Copolymer

Initially, 0.5 g (0.17 mmol) HO-PEG-NH_2_ was dissolved in 12 mL of DMSO. Separately, 1.45 g (5.85 mmol) of BzLAsp-NCA was dissolved in 8 mL of DMSO. Both solutions were degassed by bubbling argon for 1 h, and then BzLAsp-NCA solution was added dropwise via transfer needle to the HO-PEG-NH_2_ solution. The polymerization was carried out at 40 °C for 3 days under dry argon atmosphere. The reaction mixture was concentrated under vacuum, the product was precipitated in diethyl ether, dried in a vacuum oven and reprecipitated from dichloromethane/isopropanol. Yield: 75%. ^1^H NMR (600 MHz, DMSO-d_6_, *δ*, ppm): 8.18 (-αCH-N*H*-), 7.27 (C_6_*H*_5_- + -O-CH_2_-CH_2_-N*H*-), 5.00 (C_6_H_5_-C*H*_2_-), 4.60 (-αC*H*-NH- + -O*H*), 3.62 (-O-C*H*_2_-CH_2_-NH-), 3.50 (-O-C*H*_2_-C*H*_2_-O-), 3.18 (-O-CH_2_-C*H*_2_-NH-), 2.61–2.83 (-αCH-C*H*_2_-).

#### 2.3.2. End-Functionalization with LBA and Carboxyl Groups Deprotection

Initially, 0.8 g (0.087 mmol) HO-PEG-*b*-PBzLAsp, 0.063 g (0.174 mmol) LBA and 0.010 g (0.034 mmol) DPTS were dissolved in 2 mL dry DMSO under argon. Then, a solution of 0.047 g (0.226 mmol) DCC in 0.5 mL of DMSO was added. The reaction mixture was stirred at room temperature for 72 h. The solids were filtered off, the solution was concentrated, and 40 mL 1*N* NaOH was added for deprotection of benzyl groups. The solution was stirred for 1 h at room temperature, filtered, purified by ultrafiltration (MWCO 3000 Da), and the product was isolated after lyophilization. Yield: 88%. ^1^H NMR (600 MHz, D_2_O, *δ*, ppm): 4.62–4.40 (-αC*H*-NH-), 4.26–3.30 (21*H*-LBA), 3.67 (-O-C*H*_2_-C*H*_2_-O-), 2.80–2.45 (-αCH-C*H*_2_-).

### 2.4. Characterization Methods

^1^H NMR spectra were recorded in DMSO-d_6_ or D_2_O on a Bruker Avance II+ 600 MHz instrument. The average number molar mass (*M_n_*) and dispersity (*Ð*_M_) of the synthesized polymers were estimated by gel permeation chromatography (GPC) on two different systems. Shimadzu Nexera XR HPLC chromatograph with a quaternary pump, degasser, automatic injector, column heater, UV/Vis (SPD-20A) detector, differential refractive index (RID-20A) detector, 10 μm PL gel mixed-B, 5 μm PL gel 500 Å and 50 Å columns was used for the GPC analyses performed in tetrahydrofuran (THF) with a flow-rate of 1.0 mL min^−1^. The GPC analyses in DMF (+5 mmol L^−1^ LiBr) were run at 50 °C and flow rate of 1.0 mL min^−1^ on a Viscotek GPCmax system VE-2001 equipped with Viscotek TriSEC model 302 triple detector using two PLgel mixed-D columns from Polymer Laboratories. The two systems were calibrated versus polystyrene narrow molar mass standards. UV/Vis spectra were recorded on a DU 800 Beckman Coulter spectrometer. Infrared spectra were obtained from IRAffinity-1 Shimadzu Fourier Transform Infrared (FTIR) spectrophotometer with MIRacle attenuated total reflectance attachment. Transmission electron microscope (TEM) images were obtained using HRTEM JEOL JEM-2100 (200 kV) instrument equipped with CCD camera GATAN Orius 832 SC1000 and GATAN Microscopy Suit Software. The samples were prepared by depositing a drop of the aqueous PIC micelles’ dispersion onto a carbon grid and subsequent evaporation of the solvent. The images analysis was performed with ImageJ software. The size distribution of the formed PIC micelles was determined by dynamic light scattering (DLS) using a NanoBrook Plus PALS instrument (Brookhaven Instruments), equipped with a 35 mW solid-state laser operating at λ = 660 nm at a scattering angle of 90°. The particles’ hydrodynamic diameters (*d_H_*) were determined according to the Stokes–Einstein equation:

*d_H_* = *kT*/(3*πηD*) (1)

where *k* is the Boltzmann’s constant, *T* is the absolute temperature, *η* is the viscosity, and *D* is the diffusion coefficient.

The *ζ*-potentials were calculated from the obtained electrophoretic mobility by the Smoluchowski equation:

*ζ* = 4*πημ*/*ε* (2)

where *η* is the solvent viscosity, *μ* is the electrophoretic mobility, and *ε* is the dielectric constant of the solvent. The size and zeta potential measurements were carried out in an automated mode in triplicate and recorded as averages of 3 and 20 runs, respectively.

### 2.5. Polyion Complex (PIC) Micelles Preparation

The PIC micelles were prepared through a co-assembly as a result of electrostatic interactions between the oppositely charged PHEMA-*b*-PLLys and LBA-PEG-*b*-PLAsp in aqueous media. Initially, the predetermined amounts of both block copolymers were separately dissolved in a distilled water. Then, 5 mL of PHEMA-*b*-PLLys solution was added dropwise into an equal volume of LBA-PEG-*b*-PLAsp solution under stirring. The concentration of PIC micelles’ dispersion was adjusted to 1 mg mL^−1^ by adding distilled water. Polyion complex micelles with molar ratios 1:5, 1:1, and 5:1 between the charged groups of PHEMA-*b*-PLLys/LBA-PEG-*b*-PLAsp were prepared.

### 2.6. Critical Micelle Concentration (CMC) Estimation

The CMC of the oppositely charged hybrid diblock copolymers in aqueous media was estimated using the well-established dye solubilization method [39]. Briefly, UV measurements on increasing concentrations of pair oppositely charged block copolymers (0.001–1.0 mg mL^−1^) in the presence of the hydrophobic dye 1,6-diphenyl-1,3,5-hexatriene (DPH, 10 μL from 0.4 mM solution in methanol) in aqueous media were performed. The dye solubilizes into the PIC’s micellar core once the co-assembly takes place, exhibiting a characteristic spectrum with absorption maximum at 356 nm. By plotting the intensity of this maximum vs. hybrid block copolymers’ concentration, the CMC was estimated as the cross-point of the obtained two straight lines.

### 2.7. Drug Loading and In Vitro Drug Release

For the preparation of curcumin-loaded complex micelles, ethanol was used to obtain 1 mg mL^−1^ curcumin solution. Then, 1 mL from the drug solution was slowly added dropwise via syringe to 10 mL (1 mg mL^−1^) of aqueous PIC micelles’ dispersion under stirring. After the organic solvent removal on a rotary evaporator, the concentration was tuned to 1 mg mL^−1^ by adding water to the micellar dispersion in a volumetric flask. Thus, the micelles to Curc ratio in the final dispersion was 10:1 *w*/*w*.

Drug-loading efficiency (DLE) and the drug-loading capacity (DLC) of the loaded PIC micelles’ dispersions were estimated applying the following procedure: Curc-loaded aqueous micellar dispersion was filtered through a 0.45 μm membrane filter to separate the unloaded drug. The aqueous phase was removed by lyophilization, and the solid residue was redissolved in predetermined amount of acetone. The solution was subjected to UV/Vis analysis. A previously obtained value for the extinction coefficient, ε = 61,882 M^−1^ cm^−1^ (λ_max_ = 418 nm) of Curc in acetone was used to quantify the drug content into the micelles [40]. The DLE and DLC were calculated according to the following equations:

DLE (wt%) = (The mass of encapsulated Curc/The input mass of Curc) × 100(3)

DLC (wt%) = (The mass of encapsulated Curc/Total mass of the micelles) × 100(4)

The drug release kinetics (cumulative drug release vs. time) of Curc from the nanocarriers was studied by dialysis of the dispersions at 37 °C against 100 mL of distilled water containing 1% (*v/v*) Tween^®^ 20. A Spectra/Por dialysis membrane tubing (MWCO = 50,000 Da) was used to accommodate 2 mL of loaded nanocarriers aqueous dispersion. Aliquots from the release media were withdrawn periodically and analyzed by UV/Vis spectroscopy at λ_max_ value of 427 nm to estimate the Curc content.

### 2.8. Antioxidant Activity Estimation via DPPH• Radical Scavenging Assay

The DPPH*•* scavenging activity of the curcumin-loaded PIC micelles was determined using a previously described procedure with slight modifications [41]. Initially, 0.2 mM DPPH• solution was prepared in ethanol. For the analysis, 1 mL from PIC micelles dispersions in water prepared in 10–50 mg mL^−1^ concentration range were mixed with 1 mL of DPPH• radical solution. The mixtures were left in the dark at room temperature for 30 min. The absorbance values of the mixtures were measured at λ_max_ value of 517 nm by UV/Vis spectroscopy. The solution of DPPH• in ethanol/distilled water 1:1 (*v/v*) was used as a control. The DPPH• radical scavenging activity was calculated by the following equation:

% DPPH• radical scavenging activity = {(A0 − A1)/A0} × 100(5)

where A0 is the absorbance of the control, and A1 is the absorbance of the sample.

The calculated % of inhibition was plotted against the concentration, and from the graph, IC_50_ value was calculated.

## 3. Results and Discussion

### 3.1. Synthesis and Characterization of Functional Oppositely Charged Hybrid Block Copolymers

**Figure 1:**
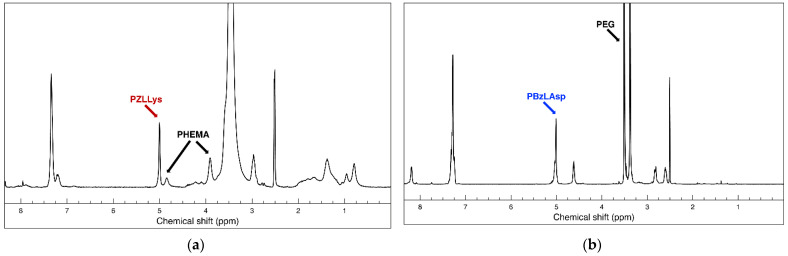
^1^H NMR (600 MHz) spectra in DMSO-d_6_ of (**a**) protected polycationic hybrid diblock copolymer PHEMA-*b*-PZLLys; (**b**) protected polyanionic hybrid diblock copolymer HO-PEG-*b*-PBzLAsp.

**Scheme 1:**
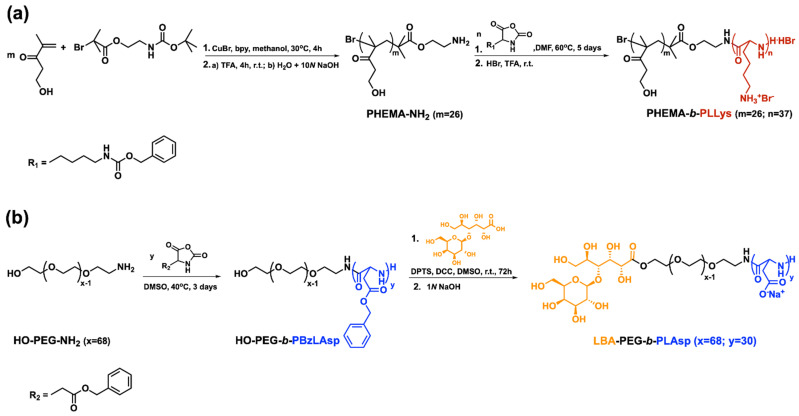
Synthetic routes toward: (**a**) positively charged PHEMA-*b*-PLLys and (**b**) negatively charged LBA-PEG-*b*-PLAsp peptide-based hybrid diblock copolymers.

A pair of hybrid block copolymers comprising two different, biocompatible hydrophilic neutral blocks and oppositely charged polypeptide blocks were obtained according to the synthetic routes presented on Scheme 1. The positively charged peptide-based hybrid diblock copolymer PHEMA-*b*-PLLys was synthesized using a specifically obtained bifunctional initiator (BAEBIB). Initially, the initiator’s bromo isobutyryl ester group was used to initiate the controlled atom transfer radical polymerization (ATRP) of the methacrylate monomer HEMA (Scheme 1a). The obtained polymer was purified, isolated and characterized. The average degree of HEMA polymerization was calculated from the ratio of integral intensities of hydroxyl protons from the PHEMA side groups at 4.85 ppm and methyl protons from the initiator’s Boc-protecting group at 1.38 ppm. The GPC analysis revealed a monomodal molar mass distribution with relatively narrow dispersity of 1.23 (Table 1). After the deprotection of the primary amine end groups, the polymer was used as a macroinitiator for the ring opening polymerization of N-carboxyanhydride of Z-protected L-lysine. The polymerization was left to proceed for 5 days at elevated temperature, and the block copolymer was purified in H_2_O/MeOH mixture. The isolated copolymer was characterized by NMR and GPC analyses. With the knowledge of the macroinitiator’s molar mass, the average degree of Z-L-lysine polymerization was estimated from the NMR spectrum in DMSO-d_6_ using the ratio of the integral areas of methylene protons of the Z-protecting groups from peptide repeating units at 5.0 ppm and the methylene protons of PHEMA side chains at 3.9 ppm (Figure 1a). The estimated average degree of polymerization was close to the target one. The block copolymer maintained the monomodal molar mass distribution with a slight increase in dispersity (Table 1).

Successful Z-L-lysine polymerization was also confirmed by FTIR spectroscopy. In the spectrum of the diblock copolymer, additional absorption bands at 1628 cm^−1^ (C=O, amide I) and 1531 cm^−1^ (N-H, amide II), characteristic of a newly formed polypeptide block, appeared (Figure S1, see details in the Supplementary Materials). The final step of this synthetic procedure involved the removal of peptide Z-protecting groups. The procedure was performed in TFA with HBr (33% in glacial acetic acid). The hybrid block copolymer was extracted in water, neutralized, purified by ultrafiltration and recovered via lyophilization. The complete removal of the protecting groups was confirmed by NMR analysis in D_2_O. There are no aromatic protons’ signals at 7.36 ppm and oxymethylene protons’ signals at 5.0 ppm characteristic for the Z-protecting groups in the product’s spectrum, whereas there is no change in the calculated degree of lysine polymerization, indicating that no degradation took place during the deprotection procedure (Figure S2a). The negatively charged hybrid diblock copolymer bearing cell targeting function (LBA-PEG-*b*-PLAsp) was synthesized according to the synthetic procedure depicted in Scheme 1b. A commercially available α,ω-heterobifunctional PEG (*M*_n_ = 3000 g mol^−1^) was used as a macroinitiator for the ring opening polymerization of freshly prepared benzyl protected L-aspartic acid N-carboxyanhydride. The polymerization was performed in DMSO as a solvent at 40 °C and was completed in three days. The product was purified and subjected to characterization. The average degree of protected amino acid polymerization was calculated from the product’s NMR spectrum as a ratio of peak intensities at 5.0 ppm characteristic for the methylene protons of the Bz-protecting groups of the peptide block and those at 3.5 ppm characteristic for the oxyethylene protons of the macroinitiator’s polyether chain (Figure 1b).

GPC analysis revealed a monomodal molar mass distribution for the diblock copolymer with dispersity of 1.3 (Table 1). The FTIR-spectrum of the diblock copolymer clearly shows the formation of the polypeptide block (Figure S3). The strong absorption bands at 1731 cm^−1^ (C=O, Asp), 1659 cm^−1^ (C=O, amide I) and 1553 cm^−1^ (N-H, amide II) confirm the successful formation of poly(L-aspartic acid) block. The next synthetic step involved the attachment of a saccharide cell-targeting function to the PEG’s hydroxyl-end group (Scheme 1b). A Steglich esterification of lactobionic acid under mild reaction conditions was performed. As a result, the PEG block of the hybrid copolymer was functionalized with a terminal cell targeting group. Upon reaction completion, the benzyl protecting groups were cleaved from the polypeptide block by the addition of 1*N* NaOH. The functional negatively charged hybrid diblock copolymer LBA-PEG-*b*-PLAsp was purified via ultrafiltration in water and was isolated after lyophilization. The complete removal of the protecting groups was confirmed from the NMR analysis in D_2_O (Figure S2b). The molar mass characteristics of the macroinitiators and oppositely charged peptide-based hybrid diblock copolymers are presented in Table 1.

### 3.2. Formation of Polyion Complex Micelles from the Synthesized Oppositely Charged Hybrid Block Copolymers

**Figure 2:**
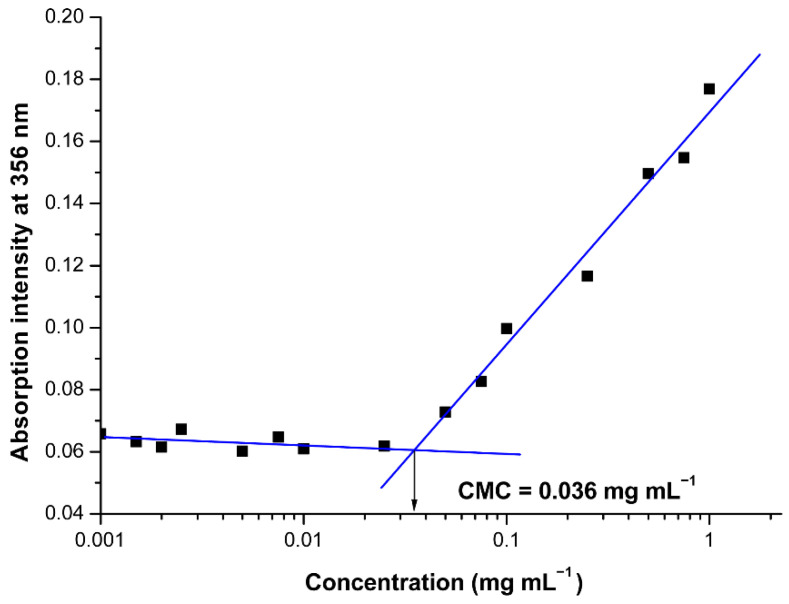
Effect of the oppositely charged block copolymers concentration on the absorption intensity of DPH at 356 nm in aqueous media for PIC micelles formation at 1:1 ionic groups’ molar ratio.

The design and synthesis of the peptide-based hybrid diblock copolymers PHEMA-*b*-PLLys and LBA-PEG-*b*-PLAsp were aimed at the construction of functional drug nanocarriers decorated with targeting ligands on the surface. The electrostatic interactions between the oppositely charged peptide segments of the block copolymers were the driving forces for the co-assembly in aqueous media. Our initial experiments clearly demonstrated the formation of thermodynamically stable PIC micelles at low concentrations upon mixing the aqueous solutions of the oppositely charged hybrid block copolymers. However, we decided to estimate the critical micelle concentration for the pair of PHEMA-*b*-PLLys and LBA-PEG-*b*-PLAsp copolymers mixed at equimolar ratio between the charged groups. A hydrophobic dye signal spectroscopic detection technique was applied due to its ability to operate at low polymer concentrations. The use of an appropriate hydrophobic dye for solubilization in the micelle’s core and detection either by fluorescence or by UV/Vis spectroscopy has already been utilized for CMC determination of amphiphilic copolymers intended for the delivery of curcumin or its derivatives [40,42]. In our case, predetermined amounts from DPH methanolic solution were injected into the aqueous polyion complex of different concentrations. As the PIC concentration increases, a sharp increase in UV absorption at 356 nm indicates the solubilization of DPH into the micelles core (Figure 2). The onset concentration of PIC micelles’ formation was estimated to be 0.036 mg mL^−1^ (1.95 μM). The estimated low CMC value, which is comparable to that of polymeric micelles, formed from amphiphilic block copolymers is an indication that the formed PIC micelles are thermodynamically stable at low concentrations, making them suitable for drug delivery applications. Furthermore, the results from the CMC evaluation experiment can be considered as a proof of concept for our intention to encapsulate hydrophobic drugs into the PIC micelles’ core. The following characterizations of the PIC micelles in aqueous media were performed at a concentration of 1 mg mL^−1^, which is a much higher value than the estimated CMC. In order to obtain PIC micelles varying in surface charge, the aqueous solutions of PHEMA-*b*-PLLys and LBA-PEG-*b*-PLAsp were mixed at three different molar ratios between the charged groups [PLLys]/[PLAsp] = 1:5 (PIC 1:5), 1:1 (PIC 1:1) or 5:1 (PIC 5:1). In all three cases, the overall PIC micelles’ concentration was kept at 1 mg mL^−1^.

**Figure 3:**
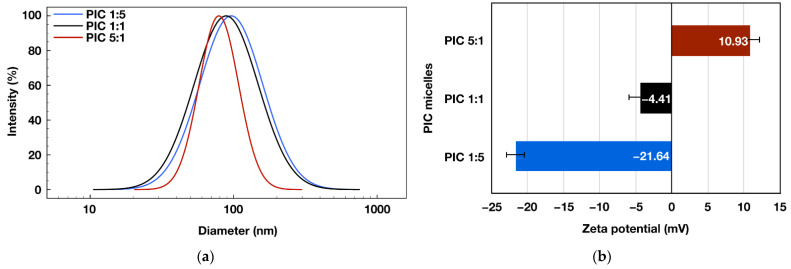
Size-distribution curves (**a**) and zeta potentials (**b**) obtained from DLS measurements of 1 mg mL^−1^ aqueous micellar dispersions of polyion complex micelles prepared at different molar ratios between the positively and negatively charged groups: PIC 1:5 (d = 95.85 nm, PdI: 0.295, ζ = −21.64 mV), PIC 1:1 (d = 90.02 nm, PdI: 0.318, ζ = −4.41 mV), PIC 5:1 (d = 79.14 nm, PdI: 0.109, ζ = 10.93 mV).

Another important parameter determining the potential application of the formed PIC micelles as drug nanocarriers is their average diameter. Thus, DLS measurements were performed on PIC micelles’ aqueous dispersions. As a result, the presence of nanosized objects was detected for all three formulations, with average diameters between 79 and 96 nm (Figure 3a). The average particles’ diameters decreased gradually with increasing the molar content of the positively charged PHEMA-*b*-PLLys copolymer into the PIC micelles. Thus, the PIC micelles prepared at [PLLys]/[PLAsp] = 5:1 exhibited the most compact structure with an average size of 79 nm and the narrowest size distribution (Table 2).

The successfully achieved control over the PIC micelles’ surface charge was demonstrated by the particles’ zeta-potential measurements. Depending on the molar ratio between the oppositely charged groups in the pair of hybrid block copolymers used for their formation, the PIC micelles are characterized with strongly negative, close to neutral or positive surface charge and zeta potentials of −21, −4 or 11 mV, respectively (Figure 3b). As a result of DLS and zeta potential measurements, it was demonstrated that by careful design and synthesis of a pair of functional oppositely charged diblock copolymers, it is possible to construct PIC micelles with adjustable surface charge and sub-100 nm sizes that might be beneficial for their potential nanomedicine applications.

**Figure 4:**
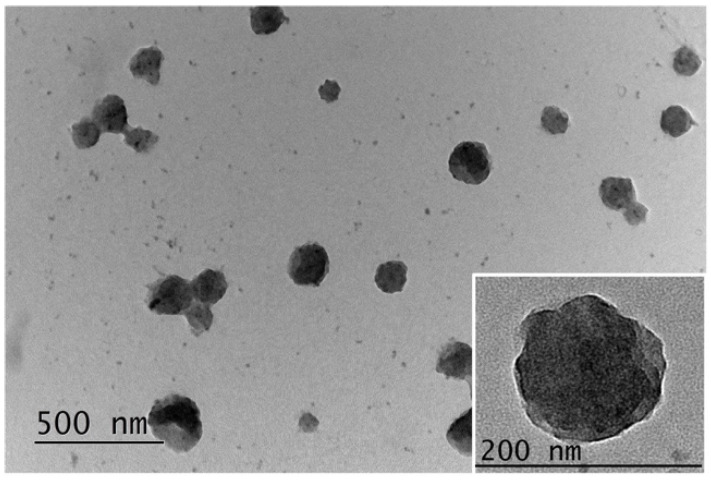
TEM images of PIC micelles formed from the oppositely charged hybrid block copolymers at molar ratio 1:1 (d = 118.07 ± 17 nm).

Transmission electron microscopy was used to visualize the PIC micelles’ morphology. The samples were prepared without staining. The images obtained show well-defined spherical PIC micelles that are dispersed mostly as individual particles (Figure 4). Their average diameters are somewhat bigger but still close to those obtained from DLS measurements. The spherical shape of the PIC micelles can be observed in more detail from the TEM image of an individual particle obtained at a higher magnification. (Figure 4, inset).

### 3.3. Hydrophobic Drug Loading into the Polyion Complex Micelles

The model hydrophobic drug curcumin (Curc) was used in the loading experiments. Curcumin (diferuloylmethane) is a natural polyphenolic substance derived from the plant *Curcuma longa*. It has demonstrated various biological properties, such as antioxidant, antibacterial, antifungal, antiviral, anti-inflammatory, anticancer, antiproliferative, proapoptotic and anti-atherosclerotic effects, leading to medicinal benefits against various forms of human illnesses [43,44]. However, Curc is characterized with low aqueous solubility, photodegradability, chemical instability, rapid metabolism and elimination that hamper its clinical application. In order to overcome the above-described drawbacks, we tested the possibility of Curc encapsulation into the formed functional PIC micelles varying in composition and surface charge. Initially, the PIC micelles were formed at a concentration of 1 mg mL^−1^ according to the already described procedure, followed by the addition of a predetermined amount of Curc solution in ethanol. After alcohol removal and final concentration adjustment, the PIC micelles to Curc ratio was 10:1 (*w*/*w*). The drug-loaded micelles were filtered to separate the free curcumin. In order to calculate drug-loading efficiency of the three types of PIC micelles, they were lyophilized, weighed and dissolved in a predetermined amount of acetone to obtain solutions with known concentrations. The amount of Curc loaded into the PIC micelles was determined quantitatively by UV spectroscopy of the acetone solutions. The calculated DLE values were between 60% and 71%. The highest DLE was established for the PIC micelles formed in a molar excess of the negatively charged hybrid block copolymer (PIC 1:5). These micelles are characterized with the largest diameters (96 nm), suggesting a larger micellar core. There is a tendency of enhancing the DLE with increasing the PIC micelle diameters (Table 2). The calculated DLC values were 6.4, 6.5 and 5.5 for PIC 1:5, PIC 1:1 and PIC 5:1 micelles, respectively. The drug-loaded PIC micelles were also subjected to DLS measurements. An approximately 10 nm increase in the average diameters was detected for all three types of PIC micelles (Figure S4a). It might be attributed to micelle core expansion as a result of the hydrophobic drug incorporation. A significant decrease in size distribution takes place after the drug-loading procedure, and all three types of PIC micelles exhibit polydispersity indices below 0.1. It might be speculated that the introduction of Curc alcohol solution to the aqueous micellar dispersions followed by the solvent removal in vacuum leads to rearrangement of the polymer chains, resulting in more uniform PIC micelles. Furthermore, the zeta potential measurements of Curc-loaded PIC micelles demonstrated that there are practically no changes in their surface charges compared to the empty analogues (Figure S4b). These results indicate that curcumin was located mainly in the interior of the PIC micelles and not on their surface. The characteristics of PIC micelles before and after drug loading are presented in Table 2.

### 3.4. In Vitro Drug Release and Antioxidant Activity Measurements

**Figure 5:**
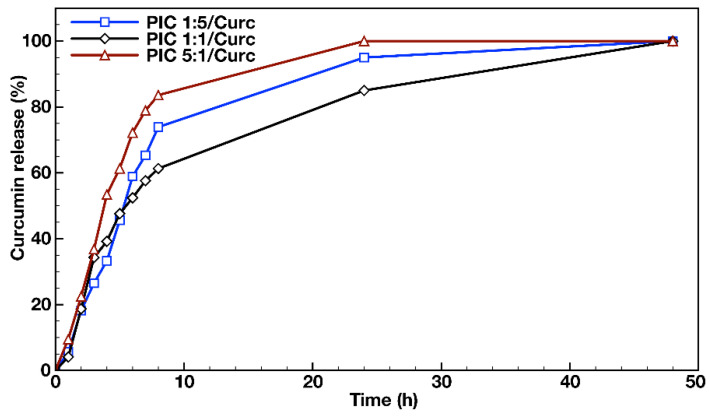
In vitro release profiles of curcumin from the PIC micelles prepared at different molar ratios between the oppositely charged groups of the hybrid block copolymers in distilled water containing 1% (*v*/*v*) Tween^®^ 20 at 37 °C.

In order to evaluate the feasibility of using PIC micelles as hydrophobic drug nanocarriers, in vitro dissolution tests were performed in an aqueous media containing 1% (*v*/*v*) Tween^®^ 20 at 37 °C using a dialysis membrane for the loaded PIC micelles’ dispersions. The released drug transferred from the PIC micelles’ dispersions via Tween^®^ 20 to the external media was quantified by UV/Vis spectroscopy at λ_max_ = 427 nm. The release media analysis of the three types of Curc-loaded PIC micelles prepared at different molar ratios between the oppositely charged hybrid diblock copolymers (PIC 1:5/Curc, PIC 1:1/Curc and PIC 5:1/Curc) revealed the achievement of a plateau with complete Curc release within 48 h (Figure 5). The three profiles were similar and biphasic, revealing a burst release in the initial stage and sustained release after the eighth hour of the evaluations. The initial burst drug release stage followed a first order-like kinetics and could be attributed to the release of the amount of curcumin that was located at the interface between the PIC micelles’ core and the hydrophilic shell. The second stage of the profile, characterized by a much slower and sustained drug release, was most likely due to the release of curcumin that was located entirely in the PIC micelles’ core as a result of its swelling with time. The DLS measurements performed on PIC micelle dispersions 48 h after their preparation revealed an average 10 nm increase in particle sizes, which was attributed to the polyelectrolyte core expansion. Although the three profiles follow the same trend, the burst effect was more pronounced in the cases of Curc loaded into the PIC micelles with charged corona and prepared in molar excess from either of the block copolymer counterparts. Thus, 84% and 74% Curc were released for 8 h from PIC 1:5 and PIC 5:1 micelles. For the same period, the released Curc from the PIC 1:1 micelles prepared at equimolar ratio between the copolymers’ oppositely charged groups and possessing neutral hydrophilic surface was 61%. Those differences between the release profiles of PIC micelles are maintained in the second stage up to the complete release at the 48th hour. It might be concluded that the PIC micelles prepared at equimolar ratios of peptide segments are more stable and better preserve the loaded hydrophobic drug under the particular experimental conditions. Moreover, the non-charged hydrophilic surface of the PIC 1:1 micelles would be beneficial for ensuring nanocarriers’ prolonged blood circulation.

**Figure 6:**
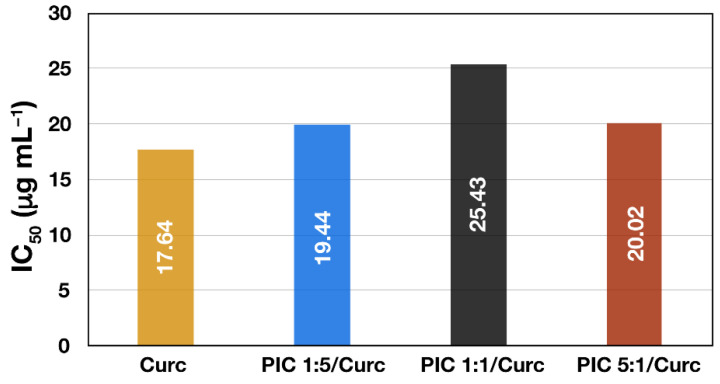
IC_50_ values of DPPH• radical scavenging activity of free curcumin and curcumin-loaded PIC micelles prepared at different ratios between the oppositely charged groups of the hybrid block copolymers.

Finally, the effect of Curc encapsulation into different types of PIC micelles on its antioxidant activity was evaluated. In addition to the drug’s improved aqueous dispersibility and stability, it is important to show that the incorporation of Curc into the nanocarrier does not negatively affect its biological properties. The DPPH• scavenging activities of Curc-loaded PIC micelles and free curcumin solubilized in ethanol/water mixture expressed as IC_50_ (μg mL^−1^) are shown on Figure 6. A high free radical scavenging activity results in low IC_50_ value, indicating that a lower concentration of the particular substance is required to reduce the activity of the probe by 50%. Thus, Curc solubilized in ethanol/water exhibited the best scavenging activity (IC_50_ = 17.64 μg mL^−1^) followed by curcumin encapsulated in surface-charged PIC micelles showing slightly lower but still similar scavenging activity—IC_50_ = 19.44 μg mL^−1^ and IC_50_ = 20.02 μg mL^−1^ for PIC 1:5/Curc and PIC 5:1/Curc micelles, respectively. The lowest DPPH• scavenging activity is demonstrated by PIC 1:1/Curc micelles (IC_50_ = 25.43 μg mL^−1^). However, all three IC_50_ values are close to that of the free drug, indicating that the antioxidant activity of the encapsulated Curc is preserved. Moreover, in aqueous media or in the blood stream where the free hydrophobic drug is practically insoluble and subjected to elimination and degradation, the curcumin-loaded PIC micelles are expected to reveal their superior antioxidant activity. The DPPH• scavenging activities of the corresponding empty PIC micelles were also evaluated. The results show weak and independent on concentration activity for all three types of empty PIC micelles. As a result, IC_50_ values of the empty micelles could not be determined. Thus, it might be concluded that the antioxidant activity of the Curc-loaded PIC micelles is mainly due to the curcumin itself, rather than the nanocarrier.

Overall, we have demonstrated the ability of specifically designed and constructed functional polyion complex micelles bearing cell-targeting ligands on the surface to encapsulate the hydrophobic natural drug curcumin. This opens the possibility to expand the application of PIC micelles in nanomedicine as carriers of hydrophobic active substances.

## 4. Conclusions

A pair of oppositely charged peptide-based hybrid diblock copolymers were synthesized, applying controlled radical and ring-opening polymerization techniques. Initially, poly(2-hydroxyethyl methacrylate)-*b*-poly(L-lysine) block copolymer was obtained and characterized followed by the synthesis of oppositely charged disaccharide-modified poly(ethylene glycol)-*b*-poly(L-aspartic acid) copolymer. The block copolymers were co-assembled into functional nanosized polyion complex (PIC) micelles at different molar ratios between the oppositely charged groups. The formed spherical core-shell PIC micelles decorated with cell-targeting ligands and with adjustable surface charge were used to encapsulate the hydrophobic natural drug curcumin. It was demonstrated that a hydrophobic drug can be successfully loaded into the PIC micelles. The nanocarriers’ drug-loading efficiency, drug-loading capacity and release profiles were evaluated. It was shown that the encapsulated drug retains its antioxidant activity. The initial evaluations of the functional PIC micelles reveal that they can be considered as potential candidates for the targeted delivery of hydrophobic drugs.

## Figures and Tables

**Table 1:** Molar mass characteristics of macroinitiators and the corresponding hybrid peptide-based diblock copolymers.

Macroinitiators				Hybrid Diblock Copolymers				
Code	*DP*_n_^a^	*M*_n_^a^ (g mol^−1^)	*Ð*_M_^b^	Code	t-*DP*_n_^c^	*DP*_n_^a^	*M*_n_^a^ (g mol^−1^)	*Ð*_M_^b^
PHEMA-NH_2_	26	3400	1.23	PHEMA-*b*-PZLLys	35	37	13,100	1.38
HO-PEG-NH_2_	68	3000	1.12	HO-PEG-*b*-PLBzAsp	35	30	9150	1.30

^a^ Number-average molar masses (*M*_n_) and degrees of polymerization (*DP*_n_), as determined by ^1^H NMR analyses. ^b^ Molar mass dispersity (*Ð*_M_), as determined by GPC analyses. ^c^ Target degree of polymerization, t-*DP*_n_ = [NCA]_0_/[NH_2_]_0_, where [NH_2_]_0_ is the active centers concentration.

**Table 2:** Characteristics of polyion complex micelles before and after drug loading.

Empty PIC Micelles				Drug Loaded PIC Micelles					
Code	d ^a^ (nm)	PdI ^a^	ζ ^a^ (mV)	d ^a^ (nm)	PdI ^a^	ζ ^a^ (mV)	DLE ^b^ (%)	DLC ^b^ (%)	IC_50_^c^ (μg mL^−1^)
PIC 1:5	95.85 ± 1.21	0.295	−21.64 ± 2.10	109.99 ± 0.92	0.077	−19.24 ± 2.00	71	6.4	19.94
PIC 1:1	90.02 ± 3.45	0.318	−4.41 ± 1.58	101.81 ± 1.83	0.077	−2.06 ± 2.35	66	6.5	25.43
PIC 5:1	79.14 ± 1.68	0.109	10.93 ± 1.31	89.35 ± 0.18	0.079	9.79 ± 1.73	60	5.5	20.02

^a^ Average micelle diameters (d), size distributions (PdI) and zeta potentials (ζ) obtained from DLS measurements. ^b^ Drug-loading efficiency (DLE) and drug-loading capacity (DLC) determined spectroscopically. ^c^ The concentration of the antioxidant required to scavenge 50% DPPH• (IC_50_) was calculated from the inhibition vs. concentration curve.

## Data Availability

Not applicable.

## Supplementary Materials

The following supporting information can be downloaded at: https://www.mdpi.com/article/10.3390/molecules27072178/s1, Figure S1: FTIR spectra of: (a) poly(2-hydroxyethyl methacrylate) macroinitiator (PHEMA-NH_2_); (b) the corresponding poly(2-hydroxyethyl methacrylate)-*b*-poly(Z-L-lysine) hybrid block copolymer (PHEMA-*b*-PZLLys). Figure S2: ^1^H NMR (600 MHz) spectra in D_2_O of deprotected: (a) poly(2-hydroxyethyl methacrylate)-*b*-poly(L-lysine) hybrid diblock copolymer (PHEMA-*b*-PLLys); (b) saccharide modified poly(ethylene glycol)-*b*-poly(L-aspartic acid) hybrid diblock copolymer (LBA-PEG-*b*-PLAsp). Figure S3: FTIR spectra of: (a) poly(polyethylene glycol) macroinitiator (HO-PEG-NH_2_); (b) the corresponding poly(ethylene glycol)-*b*-poly(*β*-benzyl-L-aspartate) hybrid block copolymer (HO-PEG-*b*-PBzLAsp). Figure S4: Size-distribution curves (a) and zeta potentials (b) obtained from DLS measurements of 1 mg mL^−1^ aqueous micellar dispersions of curcumin loaded polyion complex micelles prepared at different molar ratio between the negatively and positively charged groups: PIC 1:5/Curc (d = 109.99 nm, PdI: 0.077, ζ = −19.24 mV), PIC 1:1/Curc (d = 101.81 nm, PdI: 0.077, ζ = −2.06 mV), PIC 5:1/Curc (d = 89.35 nm, PdI: 0.079, ζ = 9.79 mV).

## Sample Availability

Samples of the compounds PHEMA-*b*-PLLys and LBA-PEG-*b*-PLAsp are available from the authors.
