# Supplementary material for: Functional Polyion Complex Micelles for Potential Targeted Hydrophobic Drug Delivery
Source: Molecules. 2022 Mar 28;27(7):2178. doi: 10.3390/molecules27072178 (PMC9000450; doi:10.3390/molecules27072178)
Supplement: Supplementary file 1 [file molecules-27-02178-s001.zip › molecules-1648911-supplementary.pdf]

Supplementary Materials

Functional Polyion Complex Micelles for Potential Targeted Hydrophobic Drug Delivery

Radostina Kalinova and Ivaylo Dimitrov*

Institute of Polymers, Bulgarian Academy of Sciences, Akad. G. Bonchev St., bl. 103-A, 1113 Sofia, Bulgaria

*Correspondence: dimitrov@polymer.bas.bg

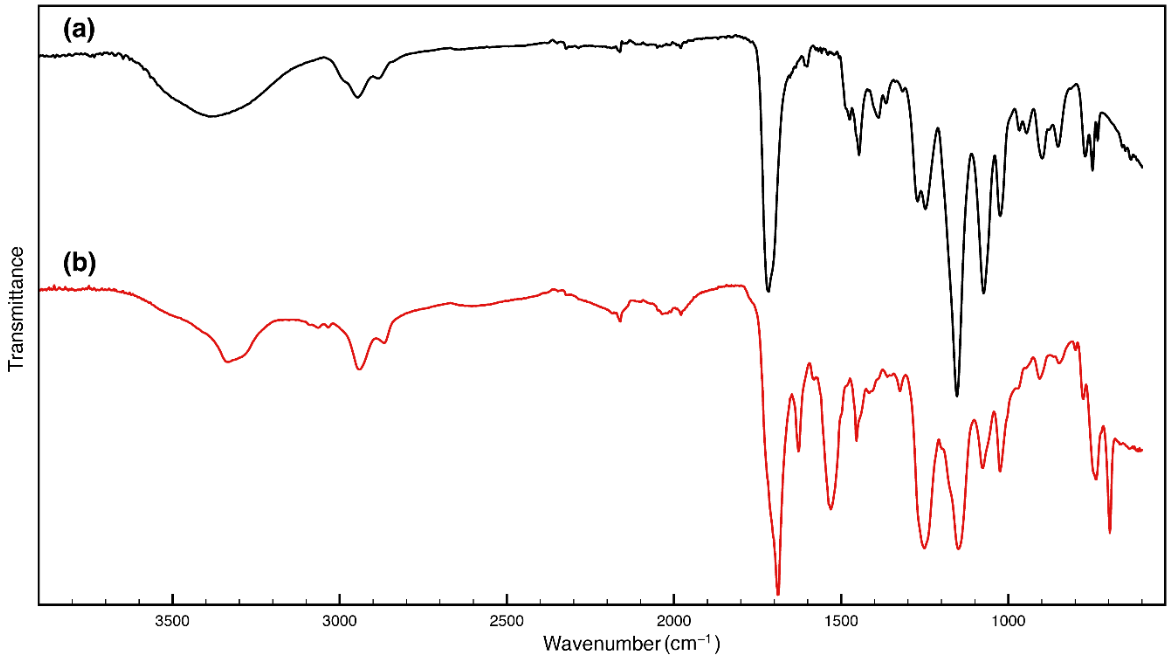

Figure S1. FTIR spectra of: (**a**) poly(2-hydroxyethyl methacrylate) macroinitiator (PHEMA-NH_2); (**b**) the corresponding poly(2-hydroxyethyl methacrylate)-*b*-poly(Z-L-lysine) hybrid block copolymer (PHEMA-*b*-PZLLys).

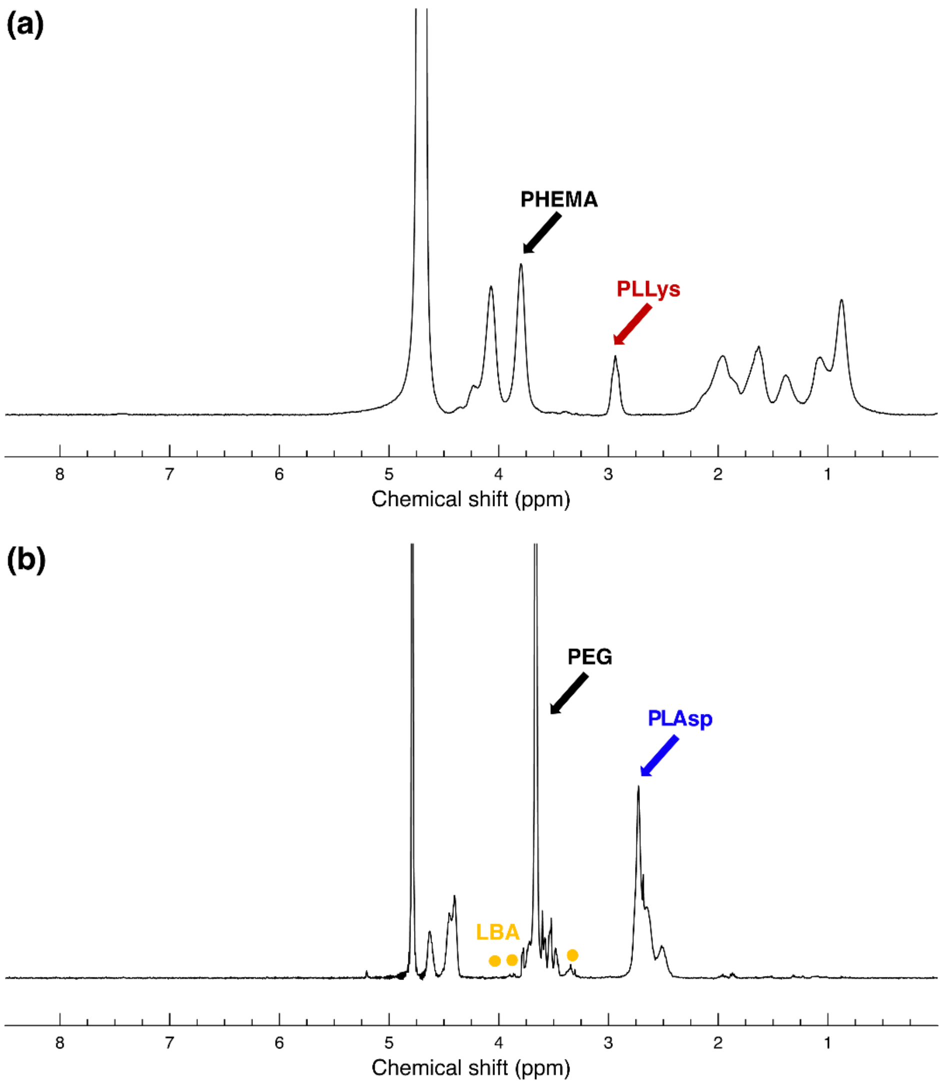

Figure S2. ^{1}H NMR (600 MHz) spectra in D_2O of deprotected: (**a**) poly(2-hydroxyethyl methacrylate)-*b*-poly(L-lysine) hybrid diblock copolymer (PHEMA-*b*-PLLys); (**b**) saccharide modified poly(ethylene glycol)-*b*-poly(L-aspartic acid) hybrid diblock copolymer (LBA-PEG-*b*-PLAsp).

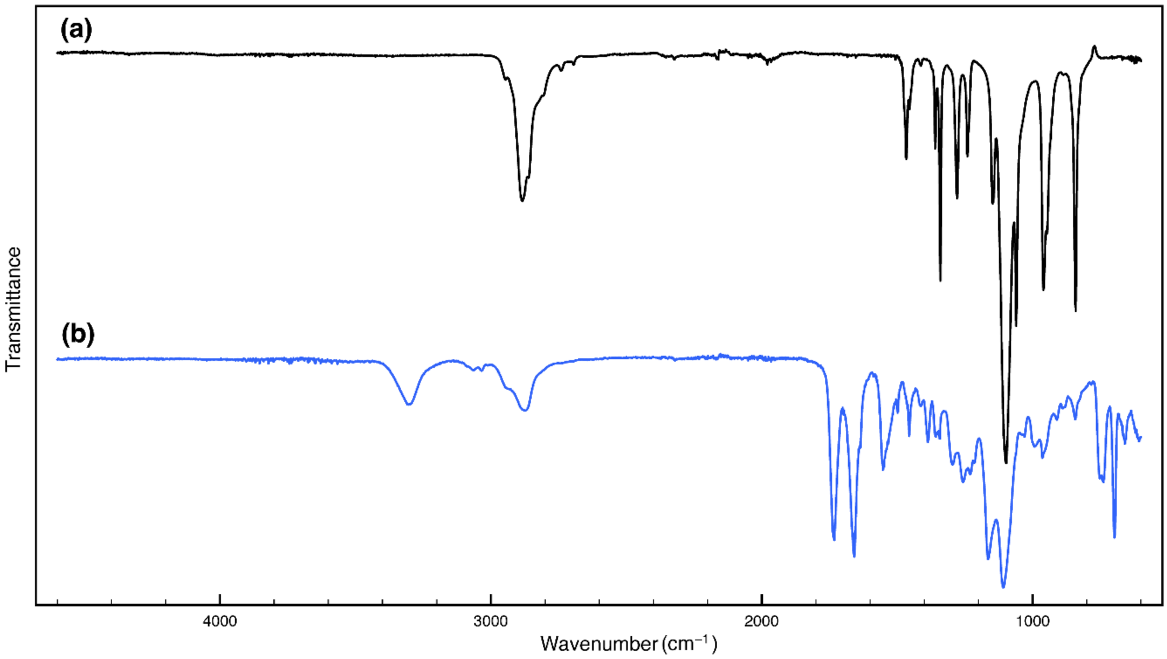

Figure S3. FTIR spectra of: (**a**) poly(polyethylene glycol) macroinitiator (HO-PEG-NH_2); (**b**) the corresponding poly(ethylene glycol)-*b*-poly(*β*-benzyl-L-aspartate) hybrid block copolymer (HO-PEG-*b*-PBzLAsp).

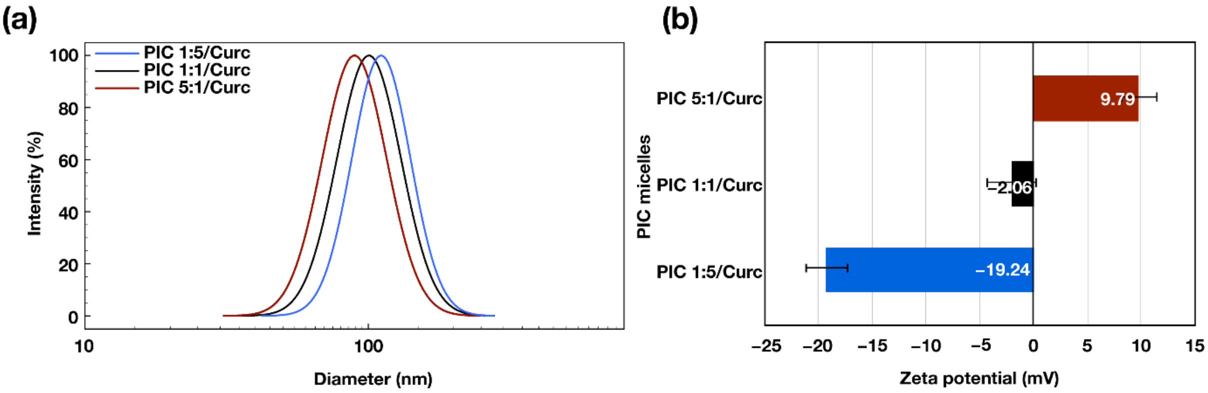

Figure S4. Size-distribution curves (**a**) and zeta potentials (**b**) obtained from DLS measurements of 1 mg mL^{-1} aqueous micellar dispersions of curcumin loaded polyion complex micelles prepared at different molar ratio between the positively and negatively charged groups: PIC 1:5/Curc (d = 109.99 nm, PdI: 0. 077, $\zeta = -19.24$ mV), PIC 1:1/Curc (d = 101.81 nm, PdI: 0.077, $\zeta = -2.06$ mV), PIC 5:1/Curc (d = 89.35 nm, PdI: 0.079, $\zeta = 9.79$ mV).
